# Genome-Wide Development of InDel-SSRs and Association Analysis of Important Agronomic Traits of Taro (*Colocasia esculenta*) in China

**DOI:** 10.3390/cimb46120796

**Published:** 2024-11-22

**Authors:** Rao Pan, Qianglong Zhu, Xinbi Jia, Bicong Li, Zihao Li, Yao Xiao, Sha Luo, Shenglin Wang, Nan Shan, Jingyu Sun, Qinghong Zhou, Yingjin Huang

**Affiliations:** 1 Jiangxi Province Key Laboratory of Vegetable Cultivation and Utilization, Jiangxi Agricultural University, Nanchang 330045, China; raopanpp@163.com (R.P.); longzhu2018@jxau.edu.cn (Q.Z.); 17379267552@163.com (X.J.); lbc012138@163.com (B.L.); zihaoli@jxau.edu.cn (Z.L.); xiaoyao1990@jxau.edu.cn (Y.X.); rosalycake@163.com (S.L.); slinyx@126.com (S.W.); shanjxau@163.com (N.S.); sunjingyu@jxau.edu.cn (J.S.); 2 College of Agronomy, Jiangxi Agricultural University, Nanchang 330045, China

**Keywords:** taro, InDel-SSR, genetic diversity analysis, association analysis

## Abstract

Taro (*Colocasia esculenta* (L.) Schott) is a tropical tuber crop whose underground corms are used as an important staple food. However, due to a lack of molecular markers, the genetic diversity, germplasm identification, and molecular breeding of taro are greatly limited. In this study, high-density InDel-SSR molecular markers covering the whole genome were developed based on the resequencing data of taro core germplasm. A total of 1,805,634 InDel-SSR loci were identified, and 219 highly polymorphic markers with an average polymorphism information content PIC value of 0.428 were screened. Furthermore, a genetic diversity analysis of 121 taro germplasm resources was conducted based on 219 markers, dividing the resources into three groups. In addition, an association analysis showed that, of the multiple InDel-SSR markers, g13.52 and g12.82 were significantly associated with leaf area and average cormel weight, respectively; the candidate genes *CeARF17* (*EVM0014444*) and *CeGA20ox* (*EVM0001890*) were related to cormel expansion; and we excavated the candidate genes *CeXXT2* (*EVM0016820*) and *CeLOG1* (*EVM0017064*), which regulate leaf development. The InDel-SSRs and candidate genes identified in this study are expected to provide important support for genetically improving and breeding new varieties of taro.

## 1. Introduction

Taro (*Colocasia esculenta* (L.) Schott) is a perennial monocot herbaceous plant from the Araceae family that is commonly cultivated as an annual crop. It is the fifth-largest root and tuber crop and the fourteenth-largest vegetable crop globally [1]. Taro has been cultivated for more than 2300 years in China and possesses a broad genetic resource, owing to its strong adaptability to arid and aquatic environments [2,3]. Based on its corm-sprouting habits, taro can be divided into three categories: kui taro, multi-cormel taro, and multi-corm taro. Kui taro has a large main corm with few unexpanded cormels and a weak sprouting ability, with the main corm serving as the primary product organ. It is mainly distributed in the coastal and southern parts of China. Multi-cormel taro has numerous expanded cormels surrounding the main corm and a strong sprouting capability; its cormels are the main product organs. It is mainly distributed in East and South China. Multi-corm taro has no significant size difference in corm or cormels that cannot be separated, thus, serves as the product organ. Multi-corm taro is primarily found in Southwestern China [4]. Compared with other crops, the large leaves and strong petioles of taro are commonly used as local specialty vegetables rich in vitamins, fiber, and protein [5,6,7]. The most important underground organ of the taro is the corm, which is rich in starch, polysaccharides, and mucoproteins. Therefore, regularly consuming taro cormels benefits gut health and enhances the immune system [8,9,10]. Leaves are the most important organs for photosynthesis and carbohydrate synthesis in taro, and traits such as leaf area and leaf shape index are closely related to the yield and quality of the corm [11,12].

Failure to bloom during growth is a common trait among many taro genetic resources in Southern China. At present, vegetative propagation is the main method for taro reproduction, with only a few reports of taro hybridization experiments [13]. The genomes of taro are large and complex. The ploidy of the three taro species is not uniform and greatly varies in genetic material [14]. Consequently, molecular biology research on taro has been limited and has lagged behind that of other major crops. Molecular markers are important tools in molecular biology research [15]. To date, several markers, including amplified fragment length polymorphisms (AFLPs), simple sequence repeats (SSRs), expressed sequence tag simple sequence repeats (EST-SSRs), and random amplification of polymorphic DNA (RAPD), have been developed and used for genetic diversity analysis and to discover regulatory genes for important agronomic traits in taro [16,17,18,19]. However, the number of molecular makers in previous genetic studies of taro is relatively small and there is a lack of genomic information on this vegetable, impeding the in-depth mining and its germplasm resources.

InDel and SSR markers are advantageous because of their wide distribution across the genome, ease of detection, and good reproducibility, making them suitable for high-density molecular marker development and widespread applications in gene mapping, association studies, and genetic linkage mapping [20,21,22,23]. The development of molecular markers that more comprehensively cover the taro genome has been enabled by the publication of the taro genome sequence. The genome contains numerous InDel variation locis, with a high repeat sequence content of 88.43% and significant genetic variation among different taro resources, making it ideal for the development of SSR and InDel molecular markers. Combining InDel and SSR markers to develop Indel-SSR markers based on a reference genome can improve marker development efficiency, reduce development costs, and provide more comprehensive genetic information.

Genome-wide association analyses have been widely applied to mine genes for important agronomic traits, yielding significant results in many monocotyledonous crops such as rice (*Oryza sativa* L.), wheat (*Triticum aestivum* L.), maize (*Zea mays* L.), and garlic (*Allium sativum* L) [24,25,26,27]. However, the mining of regulatory genes for important taro agronomic traits using genome-wide association analysis has rarely been reported. At present, only SNP markers from DArTseq have been developed for mining regulatory genes for plant height, number of leaves, and dry matter content [28]. Furthermore, few molecular marker association analyses have been conducted for yield-related traits. Therefore, developing InDel-SSR markers covering the whole genome will contribute to the mining and utilization of genes regulating important agronomic traits in taro.

This study aimed to develop a set of high-density InDel-SSR molecular markers covering the whole taro genome to analyze the genetic diversity of 121 genetic resources and mine candidate genes regulating the agronomic traits of its leaves and corms. This research not only provides insights into the genetic basis of taro agronomic traits, but also promotes identification, breeding, applications, and molecular marker-assisted breeding selection in taro.

## 2. Materials and Methods

### 2.1. Taro Germplasm Resource Cultivation

A total of 121 taro germplasm resources collected in China were utilized in this study, including 86 multi-cormel taros, 13 kui taros, 4 multi-corm taros, and 18 wild taros (Supplementary Table S2). The materials were planted in 2021 and 2022 at the Yulan Yuan Tuber Germplasm Resource Nursery (28°76′ N, 115°83′ E) of Jiangxi Agricultural University (JXAU). The field experiment was conducted in a randomized block design with three replications. Ten monocots were planted in a single row within each of the duplicates, with a plant spacing of 35 cm for multi-cormel taro, for which 50 g cormels were selected as seed taro, and kui taro and multi-corm taro were cut into chunks (about 50 g/block) as seed taro. All taro germplasm resources were uniformly cultivated and managed according to traditional methods [29].

### 2.2. DNA Extraction and Genome Resequencing

Genomic DNA was isolated from fresh leaves using an improved CTAB method during the seedling stage [30]. DNA quality was assessed using a Nanodrop spectrophotometer and agarose gel electrophoresis, and samples were stored at −80 °C for further utilization. Subsequently, high-quality DNA samples from ten taro core germplasm with phenotypes showing significantly different resources were sent to BGI for resequencing. The accessions included six multi-cormel taros (T4, T21, T22, T24, T27, and T56,), three kui taros (T46, T51, and T54), and one wild taro (T58). To carry out resequencing, 150 bp paired-end reads were used.

### 2.3. InDel-SSR Marker Detection and Polymorphism Analysis

The published taro genome (cultivar: Longxiangyu) was used as a reference, and resequencing data from ten taro materials were mapped to the reference using BWA-MEM within default parameters. The InDel loci were identified using SAMtools v1.7.2 and BCFtools v1.7.2 with the following parameters: (--min-MQ 20 --min-BQ 10) [14]. Then, 500 bp upstream and downstream sequences were extracted for each InDel locus. These sequences were employed with the following parameters to search for SSR loci near the InDel loci in the MISA v2.10: repeats of ten or more for mononucleotide motifs; repeats of five or more for dinucleotide motifs; and repeats of four or more for trinucleotide, tetranucleotide, pentanucleotide, and hexabase motifs. Furthermore, SSRs were regarded as compounds if less than 100 bp separated two SSRs. Distribution and type analyses of InDels and InDel-SSRs in the chromosomes were performed using Excel and R, with graphical figures produced using Origin 2021.

Primers for InDel-SSR markers were designed using Primer3.0 with the following parameters: primer length ranging from 16 to 24 base pairs; expected amplicon size between 100 and 300 base pairs; and a melting temperature (Tm) between 50 and 60 °C, optimizing at 55 °C, with a ≤3 °C Tm variance between primer pairs. For the subsequent polymorphism screening stage, InDel-SSR primers with a predicted product sequence containing repeat units of at least 18 base pairs in length with abundant AT bases and containing InDel loci were selected. In total, 1400 primer pairs were selected from the 14 chromosomes of the taro genome and synthesized by Shanghai Qinke Biotechnology Co., Ltd. (Shanghai, China).

Polymorphism validation was performed with ten taro accessions consistent with the sequencing samples. Primers with good amplification and high polymorphism were chosen. The PCR amplification program was set as follows: initial denaturation at 95 °C for 5 min, followed by 35 cycles of 95 °C for 1 min, 55 °C for 30 s, and 72 °C for 30 s, with a final extension at 72 °C for 10 min. The products were electrophoresed on 8% polyacrylamide gel and stained with silver. The images were gathered for subsequent examination. The electrophoretic results for the InDel-SSR markers were recorded in binary format, with bands scored as “1” and the absence of bands scored as “0”. This constructed a binary matrix, which was subsequently analyzed. Primer polymorphism and genetic diversity were calculated with GenoDive version 3.0 and Polygene 1.4, including the observed number of alleles (Na), effective number of alleles (Ne), Shannon’s diversity index (I), observed heterozygosity (Ho), expected heterozygosity (He), and polymorphism information content (PIC) [31,32].

### 2.4. Genetic Diversity Analysis

The population structure of 121 taro germplasm resources was analyzed using Structure with the Bayesian model based on the InDel-SSR binary matrix [33]. To obtain accurate parameters, the pre-set K values in Structure 2.3.4 were set from 1 to 10, and the program was run with 10,000 burn-in iterations followed by 10,000 MCMC iterations. Each K value was independently run 10 times. ΔK values were computed using Structure Harvester to determine the optimal K, and the resulting Q-matrix from Structure was consolidated with Clumpp for each K value across the multiple repetitions [34,35]. The kinship coefficients were calculated using SPAGeDi [36]. Nei’s genetic distances were calculated using NTsys 2.10e. The resulting clustering analysis and phylogenetic tree based on the above genetic distances were conducted in MEGA 7.0 with UPGMA. Principal component analysis (PCA) was conducted using the R v4.2.2 package factoextra [37,38].

### 2.5. Important Agronomic Trait Measurement

We evaluated ten agronomic traits of taro, including five leaf-related traits (leaf area, leaf length, leaf width, leaf shape index (leaf length/leaf width), and posterior segment length) and five corm-related traits (cormel number, average cormel weight, cormel diameter, cormel length, and cormel shape index (cormel diameter/cormel length)). The leaf-related traits were measured during the peak growth period from July to August, while the corm-related traits were assessed during the harvesting period from November to December. These important agronomic traits were measured using straightedges, vernier calipers, and balances. Three healthy growing plants were sampled from each block, totaling nine plants across three replicate blocks, which were randomly selected to assess the traits of each material. Phenotypic data were statistically analyzed using Excel and IBM SPSS Statistics.

### 2.6. Association Analysis and Candidate Gene Mining

An association analysis of the phenotypic and genotypic data was carried out using Tassel (version 5.2.81), and the Q matrix was used as a covariate for adjustment. Significant association loci were identified with a threshold of 0 < *p* < 0.05 [39]. Candidate genes were searched within a 1 Mb region surrounding significant InDel-SSR marker loci associated with traits. Important regulatory genes associated with important agronomic traits were preliminarily identified based on gene functional annotation in the Nr, LEGG, and GO databases and homologous functions.

## 3. Results

### 3.1. Development of Genome-Wide InDel-SSR Markers

High-quality 0.32 T sequencing data were obtained from ten taro germplasm resources. The sequencing data for each accession ranged from 23.27 to 53.70 Gb, with GC content from 41.56% to 42.49%, Q20 values ranging from 95.91% to 97.84%, and sequencing depths varying from 9.68 to 22.33X (Table 1), indicating that the resequencing data could be used for further research and marker development.

**Figure 1:**
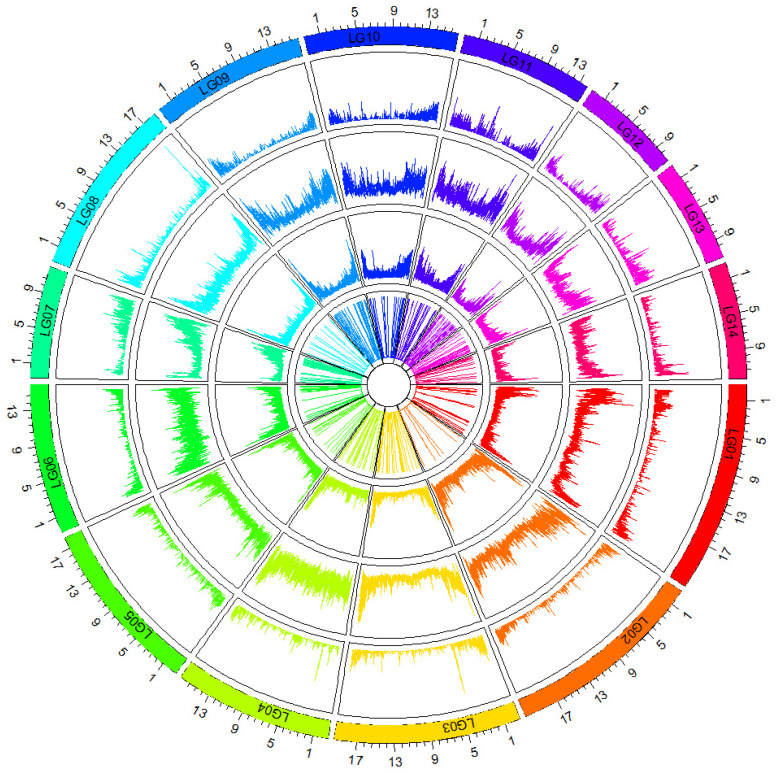
InDel and InDel-SSR distribution density map.

About 1,805,634 InDel-SSRs were identified after aligning these resequencing data to the taro reference genome, with an average of 128,974 InDel-SSRs per chromosome. The InDel-SSRs are unevenly distributed among the 14 chromosomes of the genome. Among them, InDel-SSRs were most densely distributed in Chr2 (1339.66 per Mb) and least densely in Chr3 (594.27 per Mb), with an average density across all chromosomes of 840.96 per Mb. InDel-SSRs exhibited higher density at the chromosome ends than in the central regions, with their distribution positively correlating with gene distribution (Table 2; Figure 1).

**Figure 2:**
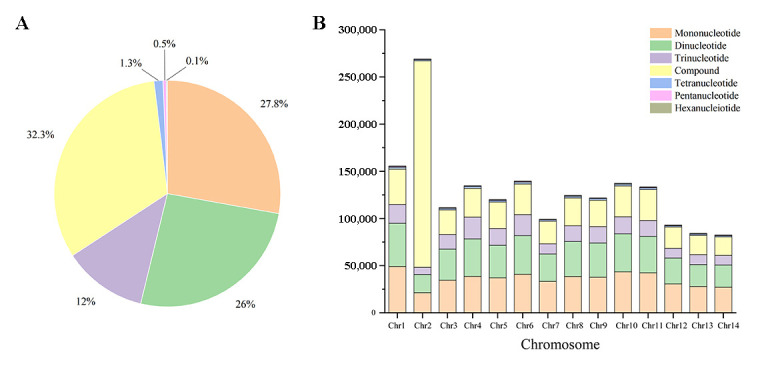
Percentage of nucleotide repeat types in InDel-SSRs in the genome (**A**) and each chromosome (**B**).

The InDel-SSR nucleotide repeat types had about 956 forms with one to six nucleotides (Figure 2A). The most frequent were the mononucleotide repeat types (A/T, C/G), about 502,598 (27.83% of the total). The dinucleotide repeat types included AT/TA, AG/CT, GA/TC, and AC/GT, with 468,736 loci (25.96%). Trinucleotide repeat types showed 216,506 loci (11.99%). Tetranucleotide repeat types showed 23,112 loci (1.28%). Pentanucleotide repeat types showed 8626 loci (0.48%). Hexanucleotide repeat types showed 2563 loci (0.14%). In addition, 583,493 compound nucleotides made up 32.32% of the total. Similar nucleotide repeat type distributions in the InDel-SSRs could be observed across all chromosomes (Figure 2B).

### 3.2. InDel-SSR Marker Detection and Polymorphism Analysis

**Figure 3:**
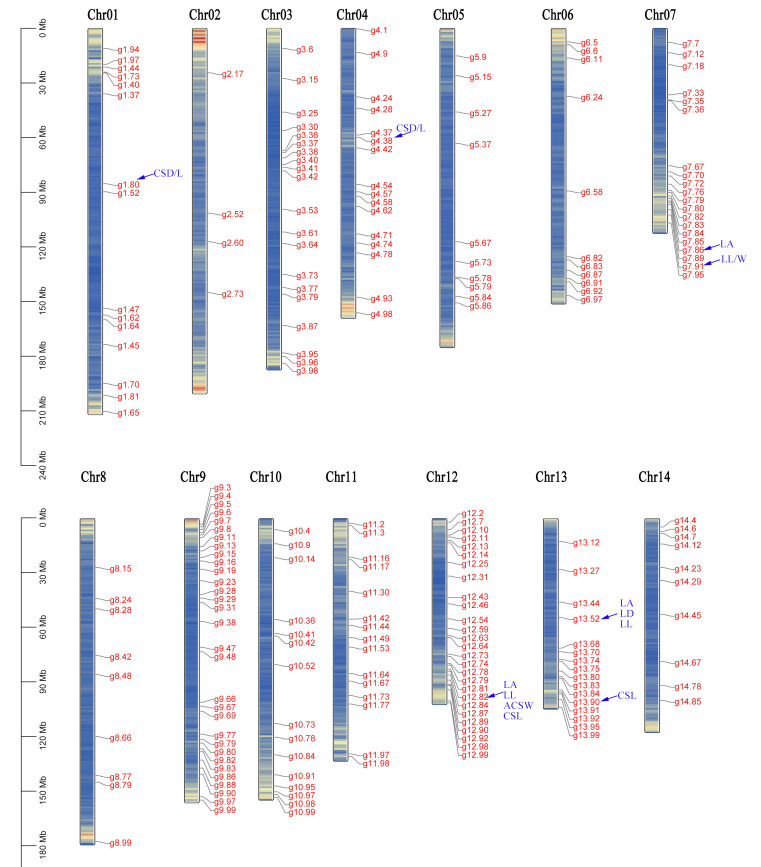
Distribution of 219 pairs of InDel-SSR polymorphic markers in taro chromosomes.

A total of 1,619,091 primer pairs were designed for the detected InDel-SSRs in the taro reference genome, representing 89.67% of the total. These primers were distributed across the 14 chromosomes, averaging 7.54 primer pairs per Mb of the sequence. The chromosome with the highest primer density was Chr2, with 12.14 primer pairs per Mb, and the lowest density was Chr3, with 5.33 primer pairs per Mb (Table 3). A total of 1400 primer pairs were synthesized, with an average of 100 primer pairs per chromosome. In total, 219 primer pairs showed significant polymorphism and good amplification after being validated with polyacrylamide gel electrophoresis. These polymorphic primers were distributed over all chromosomes and accounted for 15.64% of the primers (Figure 3, Supplementary Figure S1, and Supplementary Table S1). A total of 747 alleles (Na) were detected across all markers. The maximum number of alleles per primer pair was 6 (generated by 22 primer pairs), while the minimum was 2 (generated by 44 primer pairs), with an average of 3.41 alleles per primer pair. The number of effective alleles (Ne) ranged from 1 to 4, with an average of 1.71 per primer pair. The maximum PIC observed was 0.765, with an average value of 0.428. In total, 37.90% of primer pairs were highly polymorphic.

Ho ranged from 0 to 0.926, with an average of 0.4. He ranged from 0.006 to 0.797, with an average of 0.485. Overall, He was higher than Ho, further confirming a high level of genetic diversity in the taro population. I ranged from 0.019 to 1.649, with an average of 0.856 (Table 3). The results indicate that these primers are highly polymorphic and that the population exhibits substantial genetic diversity.

### 3.3. Genetic Diversity Analysis of Taro

The genetic structure of 121 taro germplasm resources was analyzed based on InDel-SSR molecular marker data. The optimal K value was determined using ΔK, which peaked at K = 2 (Supplementary Figure S2). Notably, the value of ΔK was second only to K = 2 when K = 3, suggesting that taro can be further divided into three groups. When K = 3, the red group consisted primarily of multi-cormel taro. The green group was dominated by wild taro and kui taro. Finally, the blue group mainly comprised multi-cormel taro and kui taro (Supplementary Figure S3).

**Figure 4:**
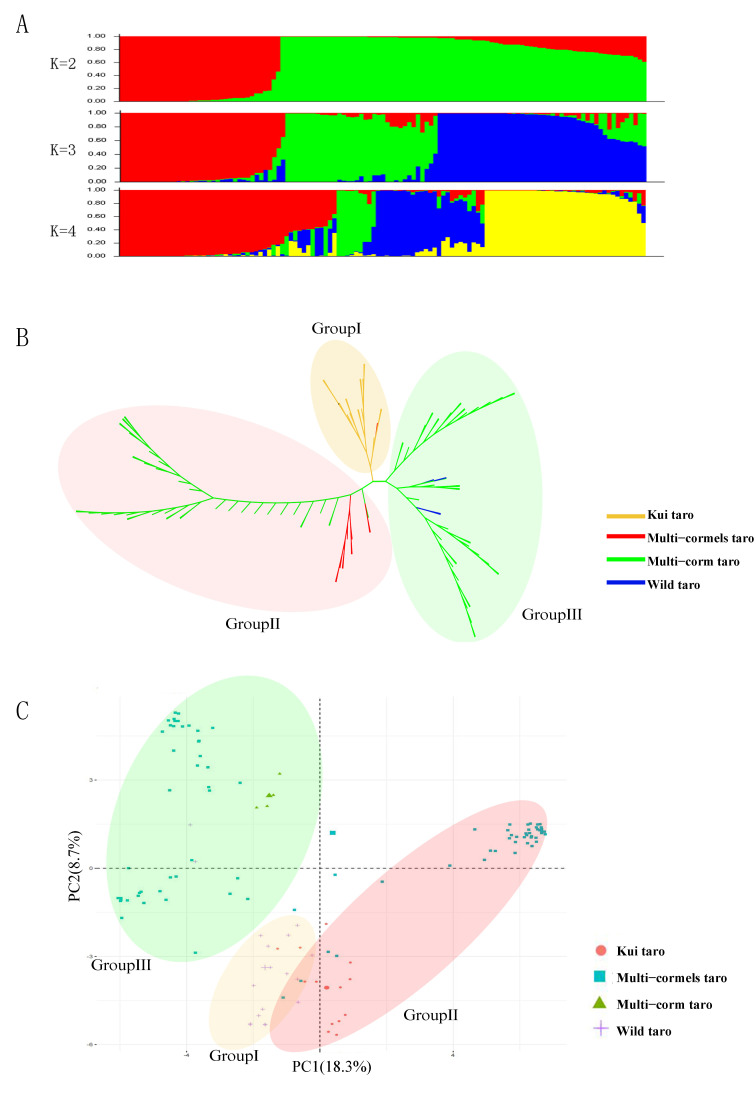
Genetic diversity analysis of taro germplasm resources. (**A**) Population structure analysis. (**B**) UPGMA-based cluster analysis of taro germplasm resources. (**C**) Principal component analysis.

**Figure 5:**
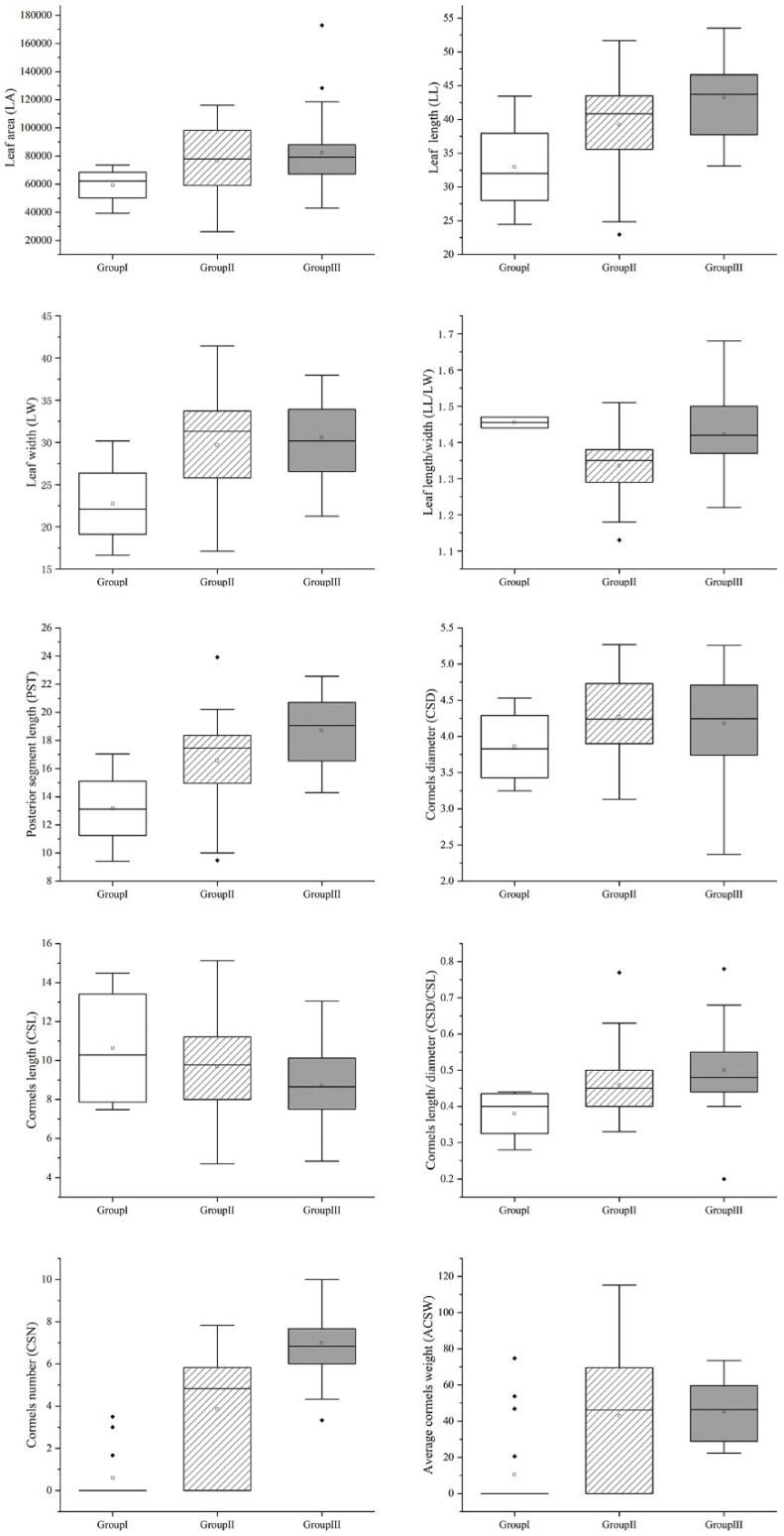
Box plot of important agronomic traits of taro.

Population genetic structure and cluster analysis of taro germplasm resources revealed that all germplasm could be optimally divided into three groups (Figure 4A,B). Group I contained 18 wild taros and 1 kui taro. Group II contained 41 multi-cormel taros and 12 kui taros. Group III contained 45 multi-cormel taros and 4 multi-corm taros. The results indicated that kui taro is genetically closer to wild taro and more distantly related to multi-cormel taro and multi-corm taro. In particular, multi-cormel taro has the greatest genetic variation and is thus divided into two separate groups, indicating that they may have different origins. PCA also supported these findings, demonstrating that kui taro is more closely related to wild taro. While multi-cormel taro is more closely related to multi-corm taro, multi-cormel taro showed significant genetic diversity (Figure 4C, Supplementary Table S3). Moreover, statistical analysis of agronomic traits among the groups revealed significant differences in leaf length, leaf shape index, posterior segment length, cormel shape index, and cormel number, indicating that plants in group I are significantly smaller than those in groups II and III in terms of leaf area, leaf width, cormel diameter, and average cormel weight (Figure 5).

### 3.4. Statistical Analysis of Important Agronomic Traits

**Figure 6:**
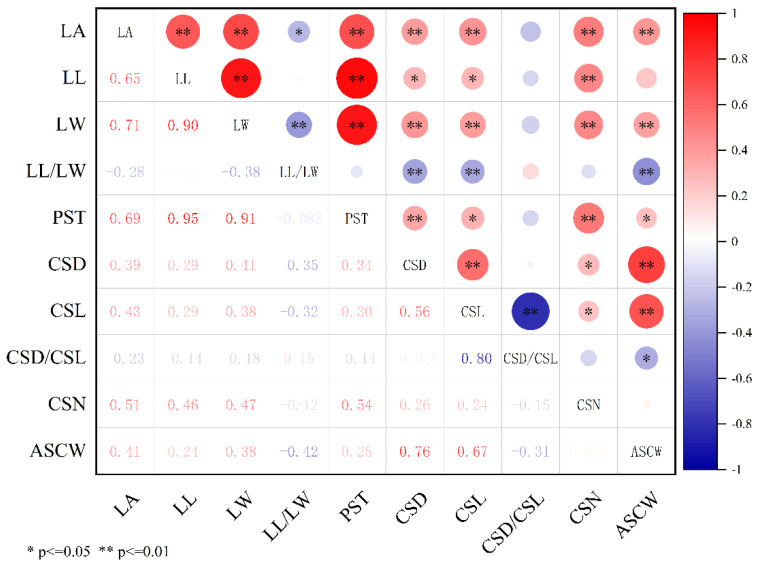
Correlation analysis of important agronomic traits of taro. * *p* ≤ 0.05, significant correlation; ** *p* ≤ 0.01, highly significant correlation.

The leaf area of the plants was surveyed in 2021, while the remaining nine important agronomic traits were surveyed in 121 taro germplasm resources over two years (2021 and 2022). Trait data were available for more than 90% of the individuals included in the study. The coefficient of variation (CV) for these traits ranged from 17.25% to 23.62%. The minimum CV was observed for leaf area in 2021 (17.25%), and the highest CV was cormel length in 2021 (23.62%). The average CV across all traits was 20.99%. However, the CV for the same traits did not differ by more than 2.4%. A one-way ANOVA showed no significant difference in traits between the two years, indicating that trait variability is relatively stable across different environments, and these experimental materials showed good genetic diversity. All ten traits followed a normal distribution (*p* > 0.05), making them suitable for subsequent association analysis (Table 4). Correlation analysis showed significant positive correlations between leaf traits and corm traits (e.g., leaf area and cormel number; leaf length and cormel number) (Figure 6).

### 3.5. Association Analysis and Candidate Gene Mining

An association analysis was conducted on 219 polymorphic markers and their correlations with leaf and corm agronomic traits, using a significance threshold set at *p* < 0.05. The general linear model (GLM) identified more associated markers than the other two association models. In total, 301 marker-trait associations were identified in 2021, whereas 181 associated markers were identified in 2022. This variation may be related to the effect of different environmental conditions on crop growth in different years. The Mixed Linear Model with Kinship (MLM (K)) and the Mixed Linear Model with Kinship and Population Structure (MLM (K + Q)) significantly reduced the number of associated markers compared with the GLM (Table 5).

Over two years, seven markers were identified with significant *p*-values in at least two different genetic models (Table 6). Four significant associated markers (g7.86, g7.91, g12.82, and g13.52) for leaf traits were identified. Particularly, the g13.52 marker was detected in association with leaf area, length, and width. The g13.52 marker, with three different band types, could distinguish leaf size, and band type I corresponded to materials with smaller leaf areas than the other band types (Supplementary Figure S4). In total, 63 genes within a 1 Mb region around g13.52 were identified and rich in cellular components, hormone signaling, and transduction, of which *EVM0016820* was homologous to *Arabidopsis thaliana* xylglucan transferase *AtXXT2*, and *EVM0017064* was involved in cytokinin synthesis. The g7.86 marker was associated with leaf area, and 80 genes were found around it that were involved in cellular components, molecular functions, and biological processes. The g7.91 marker was associated with the leaf shape index, with 75 genes around it. These genes are implicated in cellular components, biological processes, and molecular functions. Four significant associated markers (g1.80, g4.38, g12.82, and g13.90) for corm traits were found. The g12.82 marker showed significant associations with average cormel weight and cormel length. The g12.82 marker, with three different band types, could distinguish average cormel weight and length, and band type I corresponded to materials with higher average cormel weight and cormel length than the other band types (Supplementary Figure S5). In total, 54 genes around g12.82 were identified and involved in phytohormone signaling, protein synthesis and modification, transport, and metabolism, of which *EVM001444* and *EVM0001890* were related to hormone transduction. In addition, the g1.80, g4.38, and g13.90 markers were detected in association with cormel shape index and cormel length, with three, nine, and zero genes identified around these markers.

## 4. Discussion

In this study, 219 polymorphic InDel-SSR molecular markers covering the taro’s whole genome were developed based on genome resequencing data. The genetic diversity and associated analysis of 10 important agronomic traits in 121 taro germplasm resources were based on these markers. All experimental procedures were conducted according to relevant guidelines and regulations.

SSR and InDel molecular markers with the advantages of easy identification and low cost are widely distributed and relatively dense in the plant genome. These markers have been widely applied in genetic diversity analysis and genome-wide association studies [40]. In total, 1,805,634 InDel-SSR molecular markers were developed, with an average density of 840.96 markers/Mb. Furthermore, 1400 primer pairs were synthesized for polyacrylamide gel electrophoresis; 219 primer pairs could be well amplified and showed high polymorphism among 10 taro germplasms. These polymorphic InDel-SSRs were distributed in all chromosomes in this study, representing more genetic information than previous molecular markers found in taro [18,19,41]. The polymorphism rate of these primers was 15.64%, higher than that of *Rosa persica* (10.00%) and *Fagopyrum esculentum* (9.02%) [42,43]. An average of 3.41 alleles per primer pair was detected, significantly higher than the 3.20 alleles per primer pair detected by Mace in 2002 using 16 SSR markers [44]. The average PIC value of these markers was 0.428. A PIC value greater than 0.25 indicated a good degree of polymorphism. *He* was 0.485, which was higher than *Ho* at 0.4, suggesting that the markers developed were highly polymorphic and appropriate for genetic diversity analysis and genome-wide association analysis of important agronomic traits [41,45]. Elucidating the genetic diversity of plants based on molecular markers can clarify evolutionary relationships between species. In this study, 121 taro germplasm resources were optimally divided into three groups based on InDel-SSR molecular markers. Group I included 18 wild taros and 1 kui taro. Group II included 41 multi-cormel taros and 12 kui taros. Group III included 45 multi-cormel taros and 4 multi-corm taros. Wild taro and kui taro had a closer phylogenetic relationship but were genetically distant from multi-corm taro and multi-cormel taro. Multi-cormel taro showed the greatest genetic variability and was split into two groups, indicating possible different origins. Previous studies have also only confirmed that wild taro is closely related to kui taro among the 69 taro germplasm resources [46]; multi-corm taro has been clustered into multi-cormel taro, and multi-cormel taro has been divided into multiple groups based on origin and morphological characteristics [47]. Compared with these studies, ours provides a more comprehensive insight into the genetic diversity of taro.

Further analysis revealed significant increases in leaf length, leaf shape index, posterior segment length, cormel shape index, cormel diameter, average cormel weight, and cormel number in groups I to III. Additionally, leaf area, leaf width, cormel diameter, and average cormel weight in group I were significantly smaller than those in group Ⅱ and group Ⅲ. These findings indicate that the leaf size is gradually increasing and the cormel shape is gradually becoming globular (cormel shape index = 1). The trend from non-expanding to expanding cormel shapes and the conclusion regarding cormel changes in this study are supported by previous research [48,49], suggesting that the cormels of multi-cormel taro gradually expand during the domestication process. Therefore, these results further reveal the changes in leaf and cormel morphology during the domestication process and contribute to the classifying, preserving, and utilizing taro germplasm resources.

Cormels and leaves are important growth and product organs of taro; it is thus of great significance to explore the regulatory genes of their important agronomic traits; thus, a GWAS based on the InDel-SSR marker developed in this study was used to comprehensively analyze the important agronomic traits of both [50,51]. The g12.82 marker was significantly associated with average cormel weight and cormel length, and 54 genes near this marker could be taken as candidate genes. Among these genes, two hormone-related genes were confirmed via functional annotation and homology comparison: *EVM001444* (auxin response factor, ARF) and *EVM0001890* (gibberellin 20-oxidase, GA20ox). *EVM001444* (ARF) is involved in the auxin signaling pathway, and *EVM0001890* (*GA20ox*) is involved in the gibberellin signaling pathway. In particular, *CeARF17 (EVM001444*) is homologous to the Arabidopsis auxin response factor *AtARF17* (*AT5G5181*0), a gene known to inhibit adventitious root formation in Arabidopsis [52]. *OsARF17* knockout mutants exhibit significantly reduced grain length, width, and yield in rice [53]. Similarly, silencing *NtARF17* has been shown to inhibit plant growth in tobacco [54]. The *CeGA20ox* (*EVM0001890*) gene is homologous to *AtGA20ox1* (*AT4G25420*) in Arabidopsis and plays a positive regulatory role in GA biosynthesis [55]. Thus, *CeARF17* and *CeGA20ox2* could play crucial roles in cormel development and be key candidate genes for cormel weight and length. Leaf area is influenced by cell wall synthesis and remodeling, and xylan is a significant component of the cell wall [56]. The Xyloglucan-related gene *EVM0016820* (XyloGlucan 6-xylosyltransferase 2, XXT2) is near the g13.52 marker, which was significantly associated with leaf length, width, and area. This gene is homologous to Arabidopsis xylosyltransferase *AtXXT2* (*AT4G02500*). Previous reports indicate that XXT2 belongs to the xylosyltransferase family, which plays a key role in hemicellulose synthesis in plant cell walls. Knockout mutants of *AtXXT2* have shorter petioles and smaller leaves in Arabidopsis, whereas *AtXXT2* overexpression leads to larger leaf areas [57,58,59,60]. In addition, the *EVM0017064* gene (Lonely Guy, LOG) is highly homologous to Arabidopsis *AtLOG1* (*AT2G28305*) and encodes a cytokinin nucleotide 5′-monophosphate ribohydrolase. It was discovered to be near the g7.86 marker, which is associated with leaf area. The LOG enzyme participates in cytokinin biosynthesis in Arabidopsis. Its expression promotes vascular tissue differentiation in leaves (supporting leaf structure) and maintains water and nutrient transport, playing a crucial role in leaf area growth [61,62]. Thus, *CeXXT2* and *CeLOG1* are proposed as key candidate genes for regulating leaf area in taro. These results will provide a theoretical basis for further uncovering candidate genes regulating important agronomic traits in taro.

## 5. Conclusions

In conclusion, a total of 1,805,634 InDel-SSR loci were identified using the resequencing method, and 219 highly polymorphic markers were screened. The genetic diversity of 121 taro germplasm resources was analyzed and categorized into three groups based on the analysis of these markers. In addition, multiple InDel-SSR markers were found to be associated with ten agronomic traits. The genes located near these markers might be new candidates for controlling the agronomic traits of taro. These markers and candidate genes are expected to be valuable for the genetic improvement and development of new varieties of taro.

## Figures and Tables

**Table 1:** Statistics for taro resequencing information.

Accessions	DNA Concentration (ng/μL)	Total (μg)	Clean Reads	Clean Base (Gb)	GC (%)	Q20 (%)	Sequencing Depth (X)
T4	50.10	3.75	184,473,906	53.16	41.56	96.17	22.11
T21	28.30	2.29	92,251,922	27.67	41.94	97.29	11.51
T22	48.20	3.50	92,151,900	27.00	41.73	95.92	11.23
T24	66.10	4.20	184,462,146	53.70	41.78	95.91	22.33
T27	72.00	4.32	77,587,972	23.27	42.49	97.14	9.68
T46	59.60	5.24	92,208,281	27.66	42.18	97.48	11.50
T51	29.80	1.79	92,153,399	27.64	42.31	97.84	11.50
T54	38.20	3.25	92,287,632	27.68	41.85	97.58	11.51
T56	39.40	3.31	80,719,197	24.21	41.93	97.02	10.07
T58	62.30	5.36	921,09,256	27.63	42.42	97.35	11.49

Note: T4: Qujing Taro; T21: Ganyu 1; T22: Ganyu 2; T24: Ganyu 4; T27: Ganzhou Taro2; T46: Leshan Taro; T51: Quanzhou Taro; T54: Chongqing Taro1; T56: Jian Taro; T58: Lijiang Taro.

**Table 2:** Distribution of InDels and InDel-SSRs in different taro chromosomes.

Chromosome	Chromosome Length	InDel Number	InDel-SSR Number	InDel-SSR Density/Mb	InDel-SSR/InDel
Chr1	21,2136,754	404,254	155,570	733.35	38.48%
Chr2	200,729,444	561,289	268,909	1339.66	47.91%
Chr3	187,626,166	366,363	111,501	594.27	30.43%
Chr4	159,385,462	629,928	134,704	845.15	21.38%
Chr5	17,5351,756	357,483	120,041	684.57	33.58%
Chr6	151,421,878	546,905	139,596	921.90	25.52%
Chr7	112,675,773	251,974	99,058	879.14	39.31%
Chr8	179,232,514	397,430	124,390	694.01	31.30%
Chr9	156,137,564	378,460	121,793	780.04	32.18%
Chr10	154,668,131	386,074	137,204	887.09	35.54%
Chr11	133,294,484	373,230	133,426	1000.99	35.75%
Chr12	102,222,464	246,607	92,893	908.73	37.67%
Chr13	104,727,164	257,022	84,127	803.30	32.73%
Chr14	117,533,969	245,723	82,422	701.26	33.54%
Sum	2,147,143,523	5,402,742	1,805,634	840.96	33.42%
Average	153,367,395	385,910	128,974	840.96	33.42%

**Table 3:** Distribution and polymorphism analysis of 219 primer pairs in taro chromosomes.

Chromosome	Marker Mapped	Density (per Mb)	Polymorphic Primer	Na	Ne	Ho	He	PIC	I
Chr1	139,257	6.56	15	3.33	1.69	0.41	0.52	0.47	0.94
Chr2	243,778	12.14	4	3.25	1.70	0.35	0.46	0.41	0.85
Chr3	99,986	5.33	20	3.90	2.21	0.52	0.52	0.47	0.98
Chr4	120,984	7.59	16	3.69	1.78	0.45	0.53	0.48	0.97
Chr5	107,452	6.13	10	3.40	1.89	0.42	0.51	0.45	0.90
Chr6	125,518	8.29	11	3.91	1.73	0.39	0.53	0.45	0.92
Chr7	88,532	7.86	20	3.35	1.45	0.30	0.44	0.40	0.79
Chr8	111,333	6.21	9	3.67	1.61	0.37	0.48	0.43	0.87
Chr9	109,446	7.01	31	3.61	1.83	0.45	0.52	0.46	0.92
Chr10	122,851	7.94	15	2.73	1.77	0.48	0.49	0.41	0.79
Chr11	118,657	8.90	15	3.27	1.62	0.37	0.46	0.41	0.80
Chr12	82,776	8.10	27	3.11	1.48	0.30	0.40	0.35	0.70
Chr13	75,020	7.16	16	2.88	1.46	0.31	0.45	0.39	0.75
Chr14	73,501	6.25	10	3.80	1.75	0.41	0.51	0.45	0.88
All	1,619,091	105.48	219	3.42	1.71	0.40	0.49	0.43	0.86
Average	115,649	7.53	15.64	3.42	1.71	0.40	0.49	0.43	0.86

Note: Na (number of alleles); Ne (effective number of alleles); Ho (observed heterozygosity); He (expected heterozygosity); PIC (polymorphism information content); I (Shannon’s diversity index).

**Table 4:** Statistics and analysis of ten important agronomic traits of taro.

Trait	Year	Mean ± SD	Range	ANOVA	CV (%)	W	P
Leaf area (LA)/cm^2^	2021	780.43 ± 276.72	1604.49	-	17.25%	0.982	0.25
Leaf length (LL)/cm	2021	40.53 ± 7.29	34.62	0.769	21.07%	0.978	0.224
	2022	50.56 ± 5.37	24.2		22.20%	0.982	0.433
Leaf width (LW)/cn	2021	29.53 ± 5.67	25.31	0.617	22.39%	0.972	0.101
	2022	36.47 ± 4.05	19.17		21.15%	0.992	0.957
Leaf length/width (LL/LW)	2021	1.38 ± 0.1	0.55	0.329	18.67%	0.984	0.484
	2022	1.39 ± 0.09	0.48		19.08%	0.976	0.216
Posterior segment length (PST)/cm	2021	17.26 ± 3.39	16.48	0.439	20.54%	0.974	0.122
	2022	20.33 ± 2.33	10.17		22.90%	0.987	0.709
Cormel diameter (CSD)/cm	2021	4.19 ± 0.65	2.9	0.885	22.48%	0.985	0.536
	2022	4.1 ± 0.82	3.89		21.00%	0.971	0.224
Cormel length (CSL)/cm	2021	9.33 ± 2.46	10.42	0.125	23.62%	0.979	0.266
	2022	6.95 ± 1.41	6.11		23.05%	0.958	0.061
Cormel length/diameter (CSD/CSL)	2021	0.48 ± 0.12	0.61	0.083	19.22%	0.955	0.013
	2022	0.61 ± 0.16	0.82		19.62%	0.99	0.925
Cormel number (CSN)	2021	5.96 ± 2.13	11	0.714	19.39%	0.989	0.778
	2022	6.48 ± 2.16	11.33		19.02%	0.957	0.055
Average cormel weight (ACSW)/g	2021	52.48 ± 21.67	94.66	0.092	22.89%	0.959	0.019
	2022	31.89 ± 11.56	49.67		23.27%	0.983	0.654

**Table 5:** The number of significantly associated marker loci.

Trait	GLM		MLM (K)		MLM (K + Q)	
	2021	2022	2021	2022	2021	2022
Leaf area	14	-	6	-	7	-
Leaf length	47	43	9	20	8	12
Leaf width	66	17	9	17	12	18
Leaf length/width	30	26	10	8	7	0
Posterior segment length	48	26	5	12	6	12
Length/diameter of cormels	12	12	11	9	20	9
Average cormel weight	12	14	7	2	5	11
Cormel diameter	45	18	7	9	8	10
Cormel length	18	16	8	11	9	10
Cormel number	9	9	10	4	5	0
SUM	301	181	82	92	87	82

**Table 6:** Comparison of three model correlation analyses of GLM, MLM (K), and MLM (K + Q).

Trait	InDel-SSR ID	Chromosome	Year	GLM		MLM (K)		MLM (K + Q)	
				*p*-Value	R^2^	*p*-Value	R^2^	*p*-Value	R^2^
**LA**	**g7.86**	**Chr07**	2021	3.72 × 10^−5^	0.2482	9.55 × 10^−5^	0.2433	9.55 × 10^−5^	0.2433
	**g12.82**	Chr12	2021	0.0022 **	0.1191	0.0098 **	0.2433	0.0098 **	0.2433
	**g13.52**	Chr13	2021	0.0496 *	0.0397	-	-	0.0474 *	0.2433
LD	**g13.52**	Chr13	2021	2.97 × 10^−6^ **	0.3619	0.0084 **	0.5981	0.0229 *	0.6644
			2022	0.0067 **	0.2503	0.001 **	0.0478	0.0067 **	1.00 × 10^−5^
LL	**g12.82**	Chr12	2021	1.54 × 10^−4^ **	0.2581	0.0128 *	0.719	0.0145 *	0.7436
			2022	0.031 *	0.1297	0.0464 *	0.2178	0.0425 *	0.2745
	**g13.52**	Chr13	2021	2.04 × 10^−6^ **	0.2745	0.0097 **	0.7227	0.0106 *	0.7637
			2022	0.0058 **	0.1293	0.0432 *	0.4017	0.0354 *	0.5156
LL/W	g7.91	Chr07	2022	-	-	0.0121 *	0.3295	0.011 *	0.321
			2021	-	-	0.0235 *	0.662	0.0179 *	0.7222
CSD/L	g1.80	Chr01	2021	-	-	0.008 **	0.1983	0.0377 *	1.00 × 10^−5^
			2022	-	-	0.019 *	1.00 × 10^−5^	0.0245 *	1.00 × 10^−5^
	g4.38	Chr04	2021	0.036 *	0.2762	0.0395 *	0.4435	-	-
			2022	0.0056 **	0.2266	0.0212 *	1.00 × 10^−5^	-	-
ACSW	**g12.82**	Chr12	2021	0.0045 **	0.2212	0.0181 *	0.3887	-	-
			2022	0.0135 *	0.1906	0.0162 *	1.00 × 10^−5^	-	-
CSL	**g12.82**	Chr12	2021	0.0145 *	0.1984	0.0407 *	0.296	0.0191 *	0.2096
			2022	0.0122 *	0.2437	0.0085 **	1.00 × 10^−5^	0.0031 **	1.00 × 10^−5^
	g13.90	Chr13	2021	0.0456 *	0.1997	0.0323 *	0.3646	0.0436 *	0.3451
			2022	0.0358 *	0.2178	0.0439 *	0.0028	0.0358 *	1.00 × 10^−5^

Note: * These markers were significantly associated with the trait at the *p* < 0.05 level. ** These markers were significantly associated with the trait at the *p* < 0.01 level. Bold represents markers associated with at least two traits. “-” indicates that there is no detection value.

## Data Availability

The data are contained within the article and Supplementary Material.

## Supplementary Materials

The following supporting information can be downloaded at https://www.mdpi.com/article/10.3390/cimb46120796/s1: Table S1. Basic information for 219 pairs of InDel-SSR core primers. Table S2. Accessions and origins of taro varieties. Table S3: PC analysis data. Figure S1. Polyacrylamide gel electropherograms of g3.64 primer. Figure S2. Graphical depiction of the relationship between K and Δk. Figure S3. Population genetic structure of 121 taro germplasm resources (K = 3). Figure S4. The band type of g13.52 corresponds to the leaf phenotype. Figure S5. The band type of g12.82 corresponds to the corm phenotype.
