# Supplementary figures and images for: Genome-Wide Development of InDel-SSRs and Association Analysis of Important Agronomic Traits of Taro (Colocasia esculenta) in China
Source: Curr Issues Mol Biol. 2024 Nov 22;46(12):13347–63. doi: 10.3390/cimb46120796 (PMC11727045; doi:10.3390/cimb46120796)

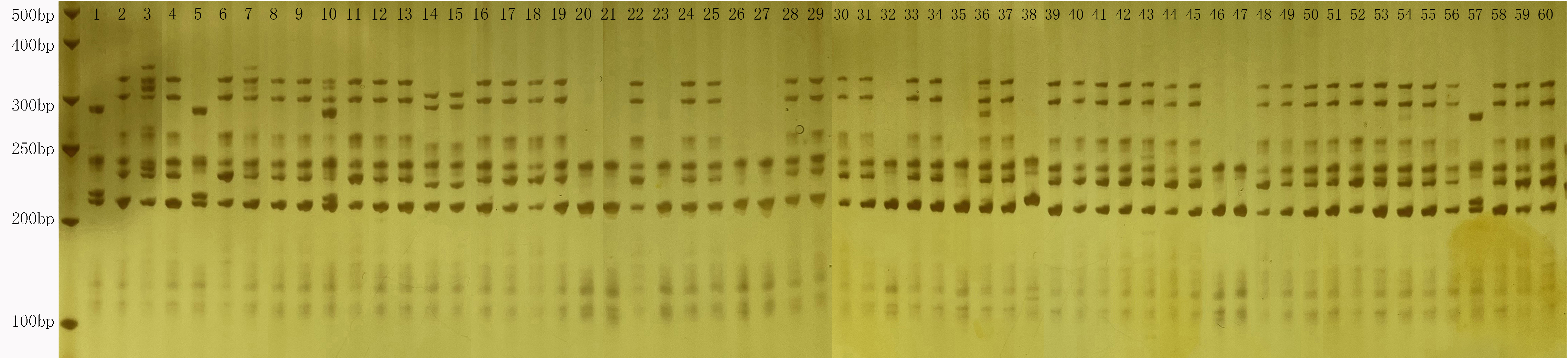

Supplement: Supplementary file 1 [file cimb-46-00796-s001.zip › Figure S1. Polyacrylamide gel electropherograms of g3.64 primer..jpg]

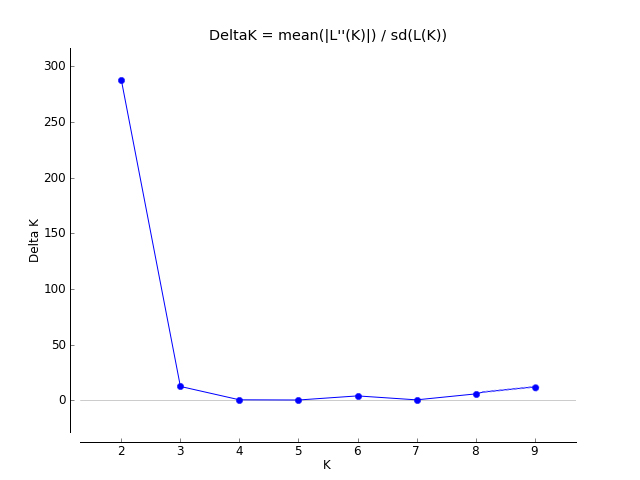

Supplement: Supplementary file 1 [file cimb-46-00796-s001.zip › Figure S2. Graphical depiction of the relationship between K and Δk.jpg]

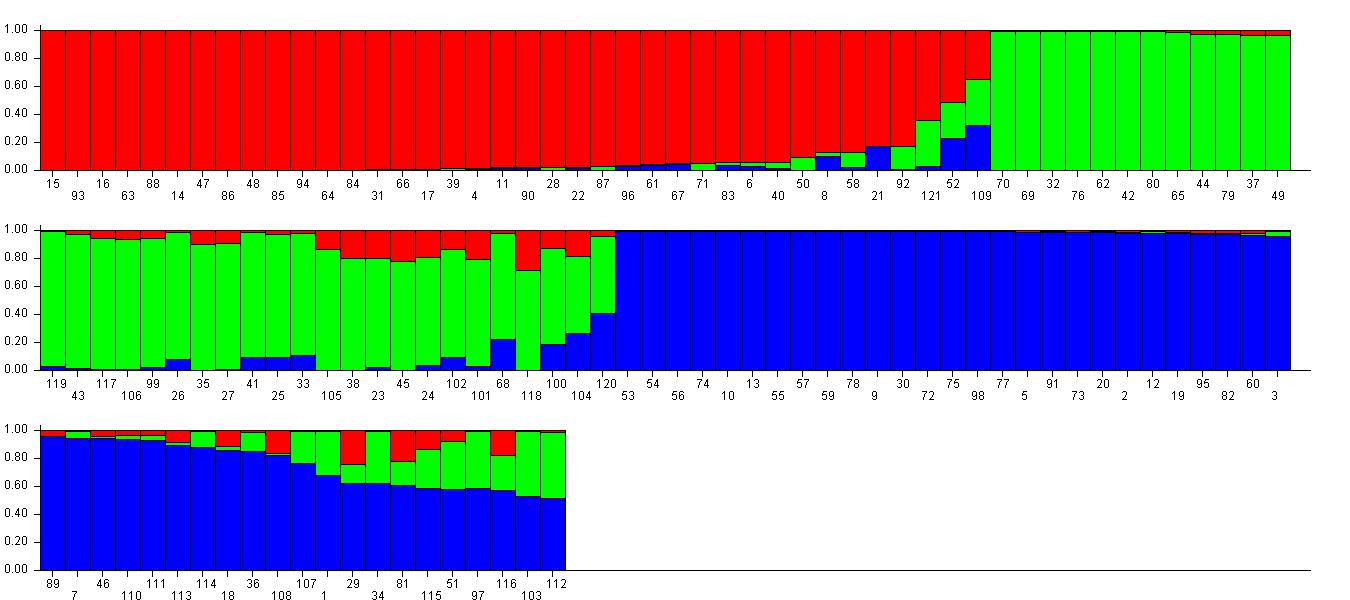

Supplement: Supplementary file 1 [file cimb-46-00796-s001.zip › Figure S3. Population genetic structure of 121 taro germplasm resources (K = 3).jpg]

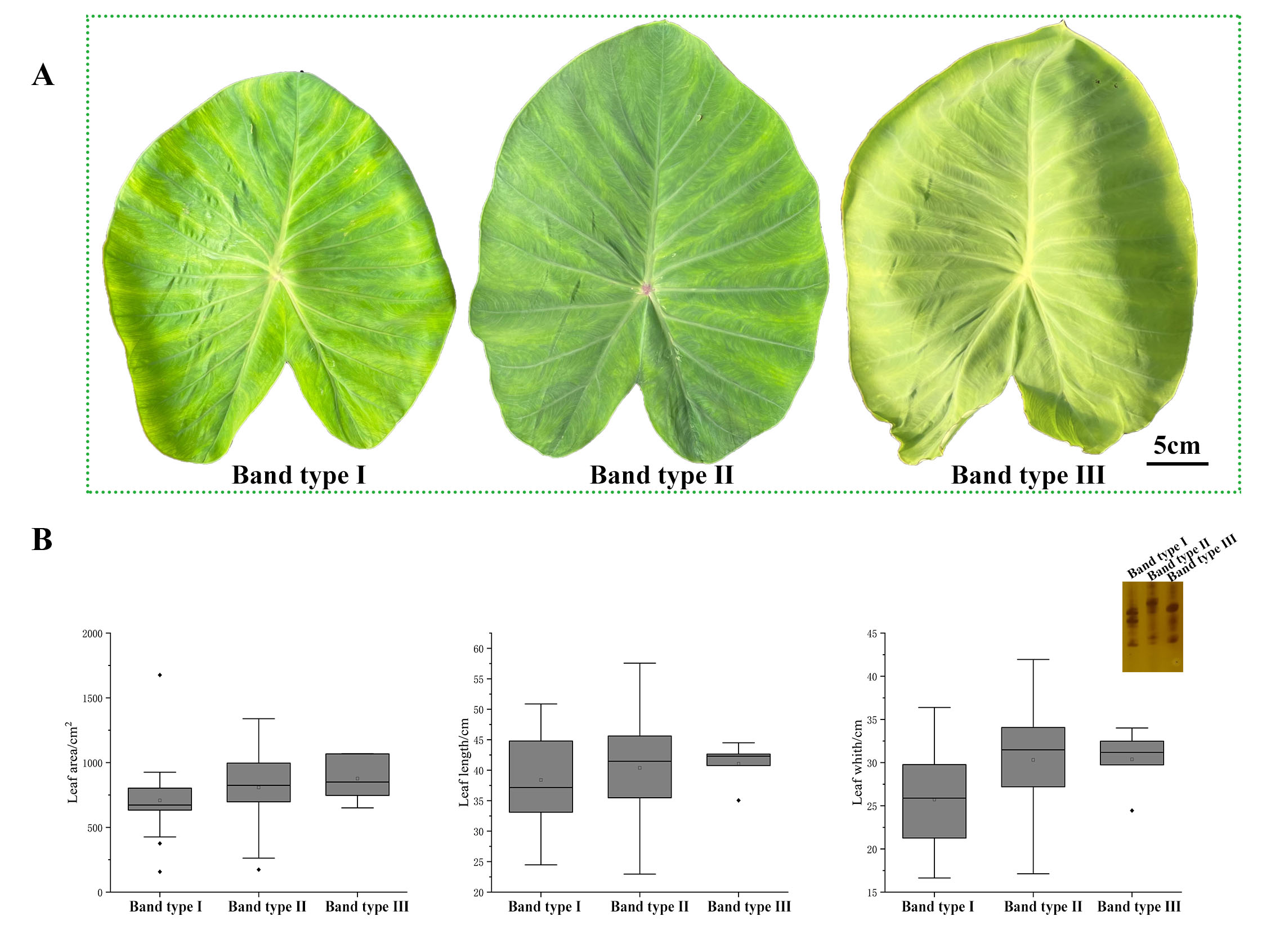

Supplement: Supplementary file 1 [file cimb-46-00796-s001.zip › Figure S4. The band type of g13.52 corresponds to the leaf phenotype..jpg]

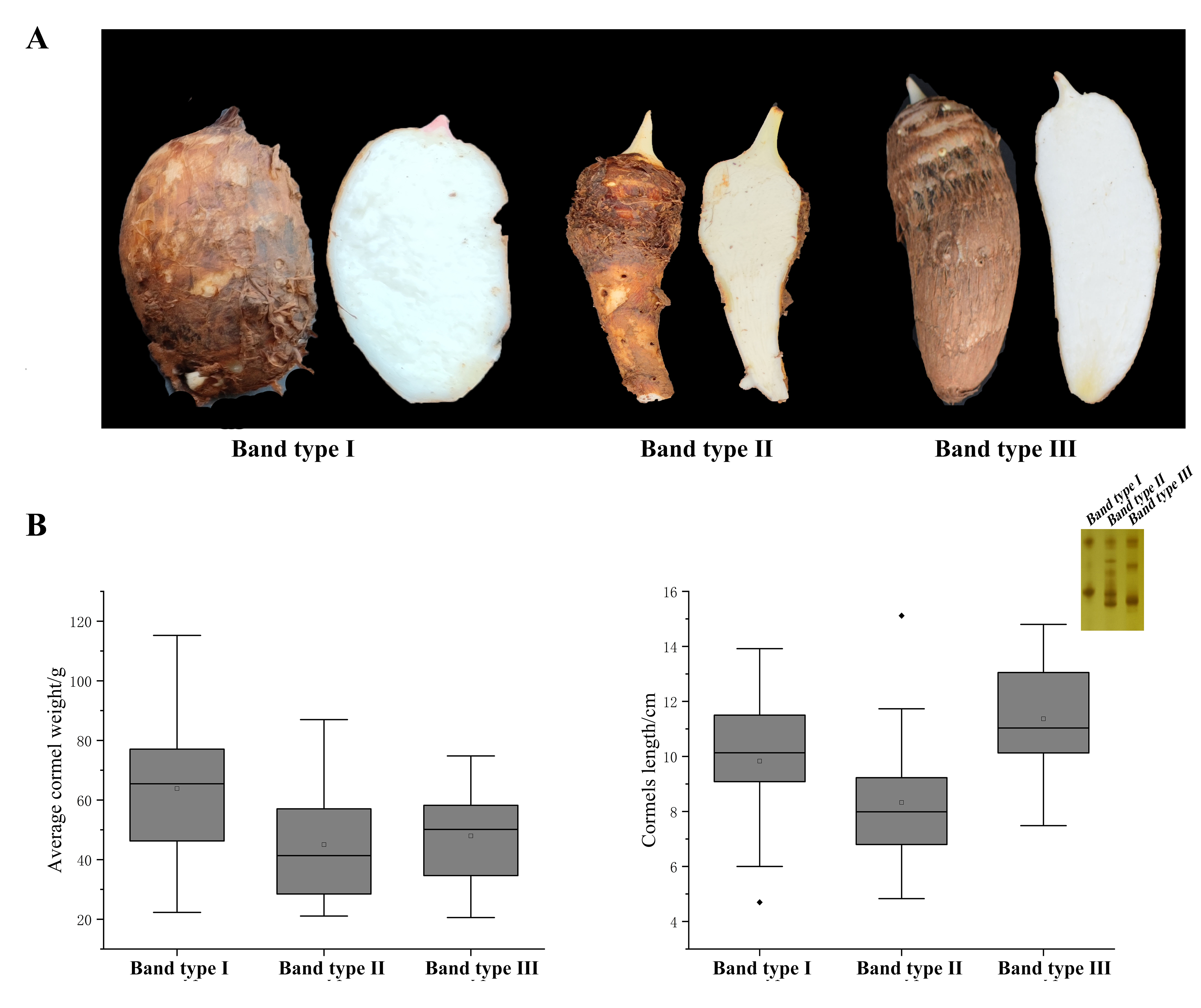

Supplement: Supplementary file 1 [file cimb-46-00796-s001.zip › Figure S5. The band type of g12.82 corresponds to the corm phenotype..jpg]
